# Single-base polymorphism of the ACE2 and TNF-alpha genes in patients with the cardiac and indeterminate forms of Chagas disease

**DOI:** 10.1016/j.bjid.2025.104574

**Published:** 2025-08-14

**Authors:** Bruna da Cruz Carvalho, Cilmery Suemi Kurokawa, Rodrigo Mattos dos Santos, Daniela Filadelfo Sanches, Simone Baldini Lucheis, Erika Alessandra Pellison Nunes da Costa

**Affiliations:** a Faculdade de Medicina de Botucatu, Universidade Estadual Paulista (UNESP), Botucatu, SP, Brazil; b Faculdade de Medicina Veterinária e Zootecnia, Universidade Estadual Paulista (UNESP), Botucatu, SP, Brazil

**Keywords:** Chagas disease, Trypanosoma cruzi, rs2074192, rs180029

## Abstract

**Introduction:**

Chagas Disease (CD) is caused by the protozoan *Trypanosoma cruzi* and is endemic to 21 Latin American countries. It is estimated that 6 to 7 million people in Latin America are infected. Clinical manifestations occur in two phases, acute and chronic. The chronic phase may present as indeterminate, cardiac, digestive, or mixed. Few studies have investigated why some infected individuals remain asymptomatic, while others develop more severe clinical forms of the disease. The present study aimed to evaluate the frequency of Single Nucleotide Polymorphisms (SNPs) in the ACE2 and TNF-alpha genes in chronic and indeterminate forms of CD and assess their association with clinical data and comorbidities.

**Methods:**

The study included 51 male patients with the indeterminate chronic forms of CD and 22 male patients with cardiac chronic forms of CD. All patients were treated at the HC-FMB/UNESP outpatient clinic. Deoxyribonucleic Acid (DNA) was extracted from blood samples and genotyped using Sanger sequencing and Restriction Fragment Length Polymorphism (RFLP) to analyze the ACE2 rs2074192 and TNF-alpha rs1800629 polymorphisms.

**Results:**

Analysis of the ACE2 rs2074192 SNP revealed no significant differences in the frequencies of the Guanine (G) and Adenine (A) alleles. Similarly, analysis of the TNF-alpha rs1800629 SNP revealed no significant differences in the frequencies of the GG, GA, and AA genotypes.

**Conclusion:**

No significant associations were found between the studied polymorphisms and the clinical forms of CD. However, further studies with larger sample sizes are needed to confirm these findings.

## Introduction

Chagas Disease (CD) is caused by the intracellular protozoan *Trypanosoma cruzi*, which is endemic in Latin America. It is present in 21 Latin American countries, affecting six to seven million people and accounting for approximately 12,000 deaths annually. It is estimated that approximately 70 million people live in areas conducive to exposure, thereby increasing their risk of infection.^1^^,^^2^

Following parasite inoculation, the acute phase of CD begins, with or without symptoms.^3^ Next, patients enter the chronic phase of the disease, in which approximately 70 % are asymptomatic and develop the indeterminate form of CD. Some patients develop a symptomatic form with cardiac or digestive tract involvement,^4^ with the cardiac form being the most frequent, affecting about 30 % of those infected.^5^^,^^6^

Despite existing studies on the pathogenesis of CD, the genetic mechanisms that influence the severity of the clinical manifestations remain poorly understood. The reason why some individuals remain with the asymptomatic form, while others progress to the symptomatic forms with varying degrees of severity is unclear. For this reason, the progression of CD may be related to immunological or inflammatory mechanisms, involved in parasite elimination, host genomic alterations, and comorbidities, especially hypertension, which involves inflammatory response mechanisms in its pathophysiology.^3^

The role of genes related to inflammation and blood pressure regulation, such as ACE2 rs2074192 and TNF-alpha rs1800629 in CD, is still poorly understood. By associating these polymorphisms with clinical data and comorbidities, especially hypertension, they can clarify the pathophysiology of CD. Understanding how host genomic changes correlate with disease forms is important to identify subgroups of patients for later clinical follow-up.^7^

Therefore, the aim of the study was to verify the frequency of the ACE2 rs2074192 and TNF-alpha rs1800629 polymorphisms in patients with cardiac and indeterminate forms of CD and to determine their association them with comorbidities.

## Materials and methods

### Subjects

This was a retrospective observational study in which 73 CD-positive male patients were evaluated. These patients received care at the General Infectious Diseases Outpatient Clinic of the Botucatu Medical School Hospital, São Paulo State University. Of these 73 individuals, 22 presented with the cardiac form of CD and 51 presented with the indeterminate form. The subjects with the chronic indeterminate and cardiac clinical forms of CD were divided into two groups for evaluation and comparison.

### Ethical considerations

This study was approved by the Research Ethics Committee of the Botucatu Medical School Hospital ‒ UNESP (Opinion n° 6.333.295). Participants gave their consent to take part in the study after receiving information and clarification on the objectives of the previous study. Then, they signed of the informed consent form.

### Inclusion criteria

This study included adult male patients in the chronic phase of CD with the cardiac and indeterminate clinical forms. The patients were treated at the General Infectious Diseases Outpatient Clinic of the Botucatu Medical School Hospital at São Paulo State University. The patients provided written informed consent and granted the use of biological samples for further studies.

### Study groups

Two study groups were formed: indeterminate and cardiac.

Patients classified as indeterminate presented positive serology in at least two methods (chemiluminescence, hemagglutination or indirect immunofluorescence) and had normal Electrocardiograms (ECG), opaque enemas, and Esophagus-Stomach-Dodenum (EED), as well as an absence of clinical symptoms. Patients with the cardiac form were individuals who, in addition to positive serology, presented with ECG changes, chest X-Ray changes and clinical symptoms such as palpitations, arrhythmias, including ventricular extrasystole, tachycardia and different degrees of heart block.

### Exclusion criteria

Female patients were excluded due to the location of the ACE2 gene on the X chromosome. Detecting the polymorphism would require analyzing both XX chromosomes of maternal and paternal origin, necessitating heterozygosity control and greater interpretive complexity. Future studies should aim to include female patients. Additionally, hormonal influences, such as contraceptive use, should be considered. Individuals who refused to participate, those with inadequate biological material, and individuals who were deceased at the time of sample collection were excluded.

### Study variables

The following the clinical data was obtained from the medical record were age (years), weight (kg), height (m), Body Mass Index (BMI), drugs for hypertension, dyslipidemia and hyperglycemia medications used by the patient, blood pressure, cholesterol values and fractions, and blood glucose values.

BMI was calculated by dividing weight in kilograms by height in meters squared (kg/m^2^). For adults aged 20- to 59-years, the classification was based on the following values: < 18.5 = Underweight, 18.5–24.9 = Normal weight, 25–29.9 = Overweight, ≥ 30 = Obese.^8^

The following values were considered in the biochemical tests for the classification of the severity of comorbidities: diabetes, total cholesterol; High Density Lipoprotein (HDL) and triglycerides. Blood glucose > 126 mg/dL; total cholesterol > 239 mg/dL, normal HDL between 40 mg/dL and 60 mg/dL, and triglycerides > 200 mg/dL.^9^^,^^10^ In addition, the continuous use of drugs to control these comorbidities was evaluated.

Isolated systolic hypertension was defined as a systolic blood pressure ≥ 130 mmHg and a diastolic blood pressure < 80 mmHg. Isolated diastolic hypertension was defined as a systolic blood pressure < 130 mmHg and diastolic blood pressure ≥ 80 mmHg, as well as the use of antihypertensive medications.^11^

### Peripheral blood collection

DNA was extracted from peripheral white blood cells. Five milliliters of peripheral blood were collected from the study participants by venipuncture in the antecubital fossa, using sterile and disposable needles and vacuum tubes. One millimiter of red cell lysis buffer RCLB (pH = 7.6, 1X, containing 10 mM Tris; 5 mM MgCl_2_ and 10 mM NaCl) was added to the leukocyte pellet which was then centrifuged at 15,000 rpm for 10 min. The supernatant was discarded, and the pellet stored at −20°C.

### Genomic DNA extraction

DNA was extracted using PureLink® Genomic DNA Mini Kits K182001 Kit (Invitrogen, Carlsbad, USA), according to the manufacturer's guidelines. The amount of DNA was determined using a NanoVue Plus spectrophotometer (GE Healthcare, Little Chalfont, Buckinghamshire, UK).

### Molecular analysis – TNF-alpha

All samples were analyzed by conventional PCR. The primers used to amplify TNF rs1800629 were the forward primer GAGGCAATAGGTTTTGAGGGCCAT and the reverse primer GGGACACACAAGCATCAAG. The PCR reaction and cycling conditions of this first reaction followed the steps described by Asghar et al.^12^ The PCR products were monitored by electrophoresis on a 2 % agarose gel, stained with 1 µL of ethidium bromide. The gel was visualized and photographed using an Ultraviolet transluminator (UVP, Upaland, CA, USA) to confirm single-band amplification.

To evaluate the TNF rs1800629, 10 μL of the amplified PCR product and 0.5 μL of the restriction enzyme Nocardia corallina (NcoI) (New England BioLabs, Boston, MA, USA ‒ R0193L) were used. Digestion was performed at 37°C in a thermocycler for 2h, followed by 65°C for 10 min. The digested samples were examined on a 3 % agarose gel under an ultraviolet transilluminator. The genotypes were differentiated by the size of their fragments: GG: 126 and 21 bp, GA: 147, 126 and 21 bp and AA: 147 bp.

### Molecular analysis – ACE2

All samples were analyzed by conventional PCR. The primers used to evaluate the ACE2 rs2074192 were the (Forward) GTAAGCCATTTCCCATCCC and primer (Reverse) ATTGTGCCACTGCCCTCTA. The PCR reaction and cycling conditions for this second reaction were performed as described by Fan et al.^11^ The PCR products were monitored by electrophoresis on a 2 % agarose gel, stained with 1 µL of ethidium bromide. The gel was then visualized and photographed using an Ultraviolet transluminator (UVP, Upaland, CA, USA) to confirm single-band amplification. An approximately 500 bp product was generated and subsequently sequenced using the Sanger method.

### Statistical analysis

Qualitative data were analyzed using either the Chi-Square test or Fischer's Exact Test, depending on the values in the contingency tables. For quantitative data, parametric analyses were performed. Student’s *t*-test was used for variables with normal distribution, and the Mann-Whitney test was used for variables with asymmetric distribution. A significance level of 0.05 was adopted for the p-value.

## Results

### Clinical and demographic data

Of the total participants, 51 presented with the indeterminate form, and 22 presented with the cardiac form.

**Fig. 1:**
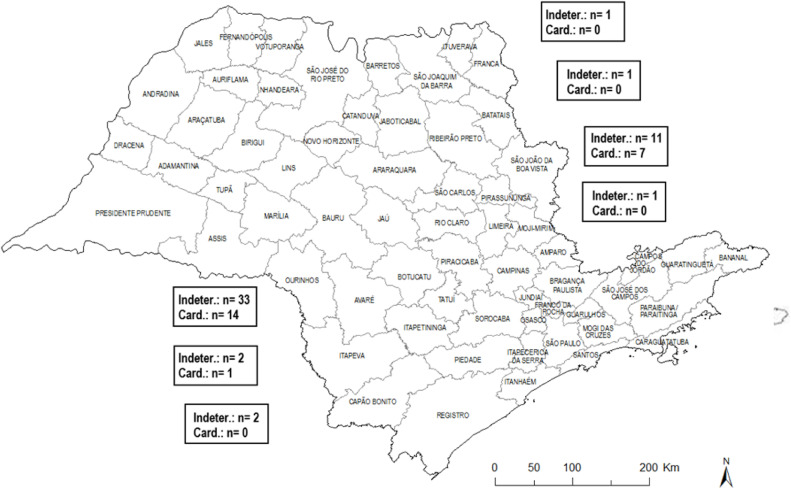
Map of the origin of patients with Chagas disease in the state of São Paulo. Indeter, Undetermined form; Card, Cardiac form; n, Number of patients belonging to each sub-region.

All 73 patients with Chagas disease were from rural areas in the state of São Paulo, Brazil. They were grouped by microregions of the State, with a focus on the subregions of Avaré (47) and Botucatu (18) (Fig. 1).

Most patients in this study, most patients resided in cities within the Avaré sub-region, 64.4 % (*n*=47) of the total of 73 patients, while 35.6 % were from other regions. Of the 51 patients with Chagas disease in the Avaré sub-region, 64.7 % (*n*=33) had the indeterminate form and 63.6 % (*n*=14) had the cardiac form. Itaí had the most patients with indeterminate forms 13.7 % (*n*=7), followed by Itaporanga 11.7 % (*n*=6); these two municipalities are part of the Avaré subregion. Among patients with the cardiac form, Bofete (Botucatu subregion) and Taquarituba (Avaré subregion) accounted for 13.6 % (*n*=3) of cases each.

There were no significant differences in BMI values between the two groups. This may be due to the outpatient follow-up provided at the Tropical Diseases Outpatient Clinic of the Botucatu Medical School Hospital, São Paulo State University (UNESP). Comorbidities are diagnosed and managed during outpatient follow-up of patients with CD (Table 1).

**Table 1:** Patients' clinical characteristics and rural professional activity.

**Variables**	**Indeterminate form** **(*n*=51)**	**Cardiac form** **(*n*=22)**	**p-value**
Age (years)	68 ± 7.92	70± 10.20	0.12^a^^,^^b^
Weight (kg)	76.13± 11.69	72.9± 13.5	0.15^a^^,^^b^
Height (m)	1.69± 0.07	1.68± 0.06	0.45^a^^,^^b^
BMI (kg/m^2^)	26.99± 3.82	25.68± 3.92	0.09^a^^,^^b^
White n (%)	41 (80.4%)	17 (77.3%)	0.33^c^^,^^d^
Rural worker n (%)	21 (41.1 %)	7 (31.8 %)	0.66^d^

kg, Kilogram; m, Meter; BMI, Body Mass Index, ratio between the individual's weight and the square of their height.

a *t*-*S*tudent.

b Mean and standard deviation.

c White vs. Non-white.

d Chi-Squared test.

The mean age of individuals with the indeterminate and cardiac forms ranged from 68- to 70-years. There was no statistical difference between the groups and all individuals were classified as elderly.

Of the evaluated comorbidities, only hypercholesterolemia showed a statistically significant difference (Table 2).

**Table 2:** Comorbidities in clinical forms.

**Variables**	**Indeterminate form** **(*n*=51)**	**Cardiac form** **(*n*=22)**	**p-value**
Hypertension	21 (41.2 %)	15 (68.2 %)	0.063^a^
Hypercholesterolemia	15 (29.4 %)	13 (59.1 %)	0.05^a^
Diabetes	12 (23.5 %)	7 (31.8 %)	0.65^a^
Obesity	8 (15.7 %)	4 (18.2 %)	1.0^b^

a Chi-Squared test.

b Fisher's exact test.

Patients were classified as obese if their BMI was greater than 30. Of the 51 asymptomatic patients, 8 (15.7 %) were obese, and of the 22 patients with the symptomatic form, 4 (18.2 %) were also considered obese. There was no statistical difference between the groups or time points evaluated (*p*>0.05). The remaining patients were considered non-obese with BMI of <30. One patient (1.9 %) had low weight for the indeterminate form, and two patients (9.1 %) had low weight for the cardiac form. Those with normal weight represented about 18 % (35.3 %) for the indeterminate form and 10 % (45.4 %) for the cardiac form. Twenty-four individuals (47.1 %) with the indeterminate form and seven individuals (31.8 %) with the cardiac form were classified as overweight.

**Fig. 2:**
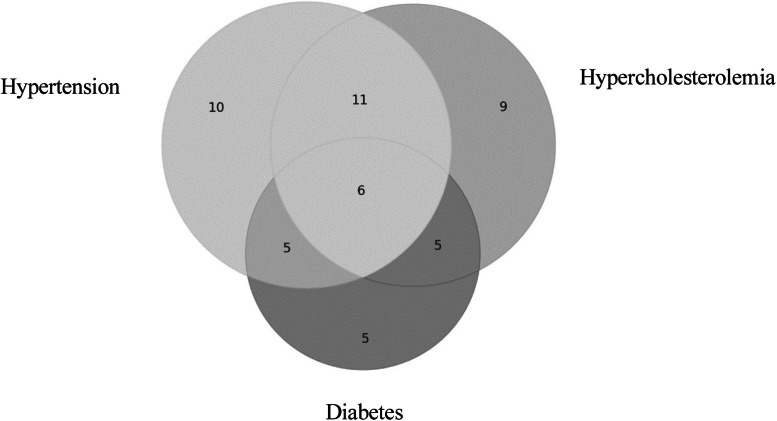
. Hypercholesterolemia diabetes diagram of the frequency of up to three comorbidities in the clinical forms.

Fig. 2 shows the distribution of up to three comorbidities among the clinical forms of CD, represented using a Venn diagram.

Table 3 presents the medications used continuously to treat Hypertension (AH), hypercholesterolemia, and diabetes/hyperglycemia. These drugs were found in the electronic medical records and helped identify each patient's comorbidities.

**Table 3:** Class of drugs.

**Variables**	**Class of drugs**	**Indeterminate form (*n*=51)**	**Cardiac form (*n*=22)**
Hypertension	Angiotensin II blockers, Alpha blockers, Beta blockers, ACE inhibitors, diuretics	20 (39.2 %)	9 (45 %)
Hypercholesterolemia	Statin	24 (47.1 %)	8 (40 %)
Diabetes/ Hyperglycemia	Biguanides, insulin, sulfonylureas, sodium-glucose transport protein inhibitors	7 (13.7 %)	3 (15 %)
No medication for comorbidities	‒	20 (39.2 %)	1 (4.5 %)

### SNP genotyping of ACE2 gene rs2074192

Regarding the ACE2 gene polymorphism genotypes between groups, the *G allele* was more prevalent in individuals with the indeterminate form (Table 4). When genotypes were analyzed within the asymptomatic and symptomatic groups, genotype G was more prevalent (Table 4). No significant differences were observed in the distribution of G and A genotypes between or within groups (*p*>0.05).

**Table 4:** Frequency of the G and A alleles of the ACE2 gene rs2074192.

**ACE2 alleles**	**Indeterminate form** **(*n*=36)**	**Cardiac form** **(*n*=7)**	**p-value**
G	24 (66.7 %)	6 (85.7 %)	
A	12 (33.3 %)	1 (14.3 %)	0.33^a^

G, Guanine, A, Adenine.

a Fisher's exact test.

There were no significant associations between genotypes and comorbidities (see Table 5). It is important to note that some samples could not be sequenced due to insufficient biological material or contamination.

**Table 5:** Frequency of the G and A alleles of the ACE2 rs2074192 gene associated with comorbidities.

**ACE2 allele**	**G**	**A**	**p-value**
Hypertension + (n)	16	6	0.87^a^
Hypertension - (n)	20	9	
Hypercholesterolemia + (n)	13	5	0.98^a^
Hypercholesterolemia - (n)	23	11	

G, Guanine; A, Adenine; (+) Presence of comorbidity; (-) Absence of comorbidity.

a Chi-Squared test.

### TNF rs1800629 genotyping

Regarding the results of the TNF-alpha gene genotypes. No significant differences were found in the distribution of GG, GA, and AA genotypes between the indeterminate and cardiac forms (Table 6, Table 7).

**Table 6:** Genotype distribution of TNF rs1800629.

**TNF-alpha genotypes**	**Indeterminate form** **(*n*=39)**	**Cardiac form** **(*n*=17)**	**p-value**
GG	19 (48.7 %)	10 (58.9 %)	
GA	17 (43.6 %)	6 (35.3 %)	0.32^a^
AA	3 (7.7 %)	1 (5.8 %)	

G, Guanine; A, Adenine.

a Chi-Square test.

**Table 7:** Frequency of GG, GA, and AA genotypes of the TNF-alpha gene and their association with comorbidities.

**TNF-alpha genotypes**	**GG**	**GA**	**AA**	**p-value**
Hypertension + (n)	11	11	3	0.34
Hypertension - (n)	18	12	1	
Hypercholesterolemia + (n)	9	10	2	0.56
Hypercholesterolemia - (n)	20	13	2	

G, Guanine; A, Adenine; (+) Presence of comorbidity; (-) Absence of comorbidity.

^a^ Chi-Squared test.

## Discussion

In this study, we analyzed 73 patients with chronic Chagas Disease (CD), divided into two groups: indeterminate and cardiac. Among Chagas patients, the prevalence of the indeterminate form was 64.4 %, while the prevalence of the cardiac form was 35.6 %, with a higher concentration in the Avaré sub-region. These findings underscore the regional significance of the disease and the challenges associated with managing its chronic manifestations.

Regarding comorbidities, hypertension was the most prevalent in both groups, followed by hypercholesterolemia and diabetes. This finding is consistent with previous studies indicating a high prevalence of these conditions in elderly patients with CD. Although hypertension was more prevalent in the cardiac form group (68.2 %), the p-value (0.063) did not reach statistical significance. However, it suggests a potential trend that requires further investigation in studies with larger sample sizes. Studies on Chagas disease patients of the same age group in Brazil have highlighted the coexistence of comorbidities in the aging process. Vizzoni et al. (2018) found that approximately 70 % of patients had at least one comorbidity. The most common comorbidities were hypertension (56 %), dyslipidemia (42 %), and type 2 diabetes (30 %).^13^ The study by Alves et al. (2009) reported an average of 2.8 comorbidities per patient. Thirty-three percent of participants had four or more chronic diseases, and the most common comorbidity was systemic arterial hypertension (57 %).^14^ Pereira et al., 2015, observed that the average number of comorbidities per patient was 2.23±1.54, with hypertension accounting for 67 %.^15^

The incidence of hypercholesterolemia among elderly Brazilians has increased considerably. This study observed a significant difference in clinical forms of hypercholesterolemia (*p*=0.05). There was also a significant difference in relation to hypercholesterolemia between the clinical forms (*p*=0.05). These high cholesterol levels directly increase the risk of cardiovascular diseases, such as myocardial infarction and stroke, in a population that is already at risk due to advanced age and other health conditions, such as hypertension and diabetes.^16^

The lack of association between the rs2074192 and rs1800629 polymorphisms and the clinical forms of CD identified in our study should be interpreted with caution. Previous research indicates that the A allele of the rs1800629 gene is linked to increased TNF-alpha production and the progression of Chronic Chagas Cardiomyopathy (CCC).^17^^,^^18^ In addition, this gene has been associated with increased susceptibility to CD in Mexican populations, emphasizing the importance of intense inflammation in the disease progression.^17^ However, this relationship was not detected in our sample, possibly due to the small number of participants and the predominance of patients undergoing continuous follow-up. This may have helped control comorbidities more effectively and attenuated clinical progression.

Regarding the genotypic versus allelic analysis of SNP rs1800629, the previous study examined the A allele in isolation. However, this study compared the GG, GA, and AA genotypes. This strategy was adopted due to the rare occurrence of the A allele in the analyzed population, which could compromise the statistical robustness of an allelic-only analysis. Nevertheless, we believe that allele-based analysis could provide valuable insights and recommend its inclusion in future research.

Regarding the rs2074192 polymorphism of the ACE2 gene, research has revealed its association with hypertension, particularly among obese men and smokers.^19^^,^^20^ Although we observed a higher prevalence of the G genotype, there were no statistical differences between the groups, nor a significant association with hypertension. This may be partially explained by the patients' constant medical monitoring, which may have reduced the clinical impact of the polymorphism on cardiovascular comorbidities.

The study had some limitations. For example, it was impossible to collect samples at two different times due to the distance between the clinic and the patients' homes. This restricted the number of cases that could be assessed. Eight samples were insufficient to evaluate the SNPs due to a lack of DNA. It was also impossible to perform the PCR reaction on ten samples due to the presence of contaminants, possibly resulting from medication use, which prevented amplification.

## Conclusion

Finally, the evaluation of polymorphisms showed no association with the severity of CD or with the comorbidities evaluated in this outpatient population. However, this link cannot be completely ruled out in patients who do not receive regular clinical follow-up or in patients with other clinical forms of the disease, such as digestive or mixed forms. Future research that includes a larger population and other clinical forms is essential to clarify the role of genetic factors in the progression of CD.

## Conflicts of interest

The authors declare no conflicts of interest.

## References

[1] Drugs for Neglected Diseases Initiative (2024). Chagas disease. https://dndial.org/doencas/doenca-de-chagas/.

[2] World Health Organization (2024). Chagas disease (also known as American trypanosomiasis). https://www.who.int/news-room/fact-sheets/detail/chagas-disease-(american-trypanosomiasis).

[3] Simões M.V., Romano M.M.D., Schmidt A., Martins K.S.M., Marin-Neto J.A. (2018). Chagas Disease cardiomyopathy. Int J Cardiovasc Sci.

[4] Santos E., Menezes Falcão L. (2020). Chagas cardiomyopathy and heart failure: from epidemiology to treatment. Rev Port Cardiol (Engl Ed).

[5] Ayo C.M., Dalalio M.M., Visentainer J.E., Reis P.G., Sippert E.Â., Jarduli L.R. (2013). Genetic susceptibility to Chagas disease: an overview about the infection and about the association between disease and the immune response genes. Biomed Res Int.

[6] Biolo A., Ribeiro A.L., Clausell N. (2010). Chagas cardiomyopathy ‒ where do we stand after a hundred years?. Prog Cardiovasc Dis.

[7] Alvarado-Arnez L.E., Batista A.M., Alves S.M. (2018). Single nucleotide polymorphisms of cytokine-related genes and association with clinical outcome in a Chagas disease case-control study from Brazil. Mem Inst Oswaldo Cruz.

[8] Diretrizes brasileiras de obesidade 2016. Associação Brasileira para o Estudo da Obesidade e da Síndrome Metabólica, 2016.

[9] Rodacki M., Cobas R.A., Zajdenverg L., Silva Júnior W.S., Giacaglia L., Calliari L.E. (2024).

[10] Bundy J.D., Mills K.T., He J. (2019). Comparison of the 2017 ACC/AHA hypertension guideline with earlier guidelines on estimated reductions in cardiovascular disease. Curr Hypertens Rep.

[11] Fan Z., Wu G., Yue M., Ye J., Chen Y., Xu B. (2019). Hypertension and hypertensive left ventricular hypertrophy are associated with ACE2 genetic polymorphism. Life Sci.

[12] Asghar T., Yoshida S., Kennedy S., Negoro K., Zhuo W., Hamana S. (2004). The tumor necrosis factor-alpha promoter -1031C polymorphism is associated with decreased risk of endometriosis in a Japanese population. Hum Reprod.

[13] Vizzoni A.G., Varela M.C., Sangenis L.H.C., Hasslocher-Moreno A.M., Brasil P.E.A.A., Saraiva R.M. (2018). Ageing with Chagas disease: an overview of an urban Brazilian cohort in Rio de Janeiro. Parasit Vectors.

[14] Alves R.M., Thomaz R.P., Almeida E.A., Wanderley J.S., Guariento M.E. (2009). Chagas' disease and ageing: the coexistence of other chronic diseases with Chagas' disease in elderly patients. Rev Soc Bras Med Trop.

[15] Pereira N.S., Queiroga T.B.D., Nunes D.F., Andrade C.M., Nascimento M.S.L., Do-Valle-Matta M.A. (2018). Innate immune receptors over expression correlate with chronic chagasic cardiomyopathy and digestive damage in patients. PLoS Negl Trop Dis.

[16] Izar M.C.O., Giraldez V.Z.R., Bertolami A., Santos Filho R.D., Lottenberg A.M., Assad M.H.V. (2021). Atualização da Diretriz Brasileira de Hipercolesterolemia Familiar –2021. Arq Bras Cardiol.

[17] Rodríguez-Pérez J.M., Cruz-Robles D., Hernández-Pacheco G., Pérez-Hernández N., Murguía L.E., Granados J. (2005). Tumor necrosis factor- alpha promoter polymorphism in Mexican patients with Chagas' disease. Immunol Lett.

[18] Alvarado-Arnez L.E., Batista A.M., Alves S.M., Melo G., Lorena V.M.B., Cardoso C.C. (2018). Single nucleotide polymorphisms of cytokine-related genes and association with clinical outcome in a Chagas disease case-control study from Brazil. Mem Inst Oswaldo Cruz.

[19] Hamet P., Pausova Z., Attaoua R., Hishmih C., Haloui M., Shin J. (2021). SARS-CoV-2 receptor ACE2 gene is associated with hypertension and severity of COVID-19: interaction with sex, obesity, and smoking. Am J Hypertens.

[20] Pan Y., Wang T., Li Y., Guan T., Lai Y., Shen Y. (2018). Association of ACE2 polymorphisms with susceptibility to essential hypertension and dyslipidemia in Xinjiang, China. Lipids Health Dis.

